# Prognostic and clinicopathological significance of neutrophil-to-lymphocyte ratio in patients with oral cancer

**DOI:** 10.1042/BSR20181550

**Published:** 2018-12-11

**Authors:** Yun Yang, Rongxun Liu, Feng Ren, Rui Guo, Pengfei Zhang

**Affiliations:** 1 School of Basic Medical Sciences, Xinxiang Medical University, Xinxiang, China; 2 Henan Collaborative Innovation Center of Molecular Diagnosis and Laboratory Medicine, Xinxiang, China; 3 College of Biomedical Engineering, Xinxiang Medical University, Xinxiang, China

**Keywords:** meta-analysis, neutrophil- to-lymphocyte ratio, oral cancer, prognosis

## Abstract

**Objectives:** Many studies have examined the prognostic significance of the neutrophil-to-lymphocyte ratio (NLR) in oral cancer; however, the results are contradictory. We, therefore, conducted a meta-analysis aiming to clarify the prognostic value of the NLR in oral cancer patients. **Methods:** A literature search was conducted in the PubMed, Web of Science, and Embase databases. Stata version 12.0 was used for statistical analysis. **Results:** A total of 14 studies with 3216 patients were finally included. The results indicated that a high NLR was significantly associated with worse DFS (*n*=10, HR = 1.73, 95% confidence interval [CI] = 1.44–2.07, *P*<0.001). Similar results were observed for overall survival (OS) (*n*=9, HR = 1.61, 95% CI = 1.39–1.86, *P*<0.001). Moreover, a high NLR was also correlated with lymph node metastasis (*n*=7, odds ratio [OR] = 1.62, 95% CI = 1.32–1.98, *P*<0.001), advanced tumor stage (*n*=7, OR = 2.63, 95% CI = 2.12–3.25, *P*<0.001), T stage (*n*=6, OR = 3.22, 95% CI = 2.59–4.01, *P*<0.001), tumor differentiation (*n*=5, OR = 1.48, 95% CI = 1.03–2.11, *P*=0.033), and perineural invasion (*n*=4, OR = 1.83, 95% CI = 1.4–2.39, *P*<0.001). However, an elevated NLR was not correlated with gender. **Conclusion:** This meta-analysis showed that the NLR might be a potential independent prognostic factor in patients with oral cancer.

## Introduction

Oral cancer is a malignant neoplasia occurring in the lip or oral cavity, and is traditionally defined as squamous cell carcinoma (SCC) because 90% of cancers in the dental area originate from squamous cells [1]. Oral cancer is within the top 10 diagnosed cancers, and it is more prevalent in Asian countries, especially Southeast Asia [2]. Unfortunately, the incidence rate of oral cancer in Asia is still rising [2]. Despite progress in research and treatment in the past few decades, the survival of oral cancer patients has not substantially improved [3]. Prognostic biomarkers are essential for treatment because they can provide valuable information for prognosis prediction and therapeutic regimen selection. A variety of potential prognostic markers of oral cancer has been investigated and reported [3]. However, tissue specimens are needed to detect these molecular markers. In addition, due to the lack of follow-up studies, the clinical significance of these potential biomarkers cannot be guaranteed [3]. As a result, convenient and non-invasive prognostic biomarkers for oral cancer are still urgently needed.

It is well established that cancer has a close connection with inflammation [4]. Inflammatory responses play important roles at each step of tumor development, including initiation, progression, and metastasis [5]. The neutrophil-to-lymphocyte ratio (NLR) is an important hematological parameter that reflects inflammatory responses. Furthermore, the NLR is a readily available and inexpensive biomarker. The NLR has been reported to be a significant prognostic marker for a number of malignancies, including colorectal cancer [6], bladder cancer [7], breast cancer [8], ovarian cancer [9], and non-small-cell lung cancer [10]. Recently, many studies have also explored the prognostic and clinical significance of the NLR in oral cancer; however, the results have been inconclusive [11–16]. Therefore, to further verify the prognostic role of the NLR in oral cancer, we conducted a meta-analysis by pooling data from relevant studies.

## Materials and methods

### Search strategy

A systemic and comprehensive literature search was performed in the PubMed, Web of Science, and Embase databases up to November 2018. The following key terms were used: ‘oral cancer’ or ‘oral squamous cell carcinoma’ or ‘OSCC’ and ‘neutrophil-to-lymphocyte ratio’ or ‘NLR’. This meta-analysis was performed according to the guidelines of the Preferred Reporting Items for Systematic Reviews and Meta-Analyses Statement (PRISMA) [17].

### Selection criteria

The inclusion criteria were as follows: (1) patients with a histologically confirmed diagnosis of oral cancer; (2) studies evaluated the association between the NLR and survival outcomes or clinicopathological characteristics in oral cancer; (3) a definite cut-off value of the NLR was used to stratify patients; (4) sufficient information was provided to allow the calculation of HRs and 95% confidence intervals (CIs) for survival analysis [18]; and (5) full-text articles published in English. The exclusion criteria were: (1) reviews, conference abstracts, letters, or case reports; (2) multiple cut-off values of the NLR were used; (3) animal studies; and (4) duplicate studies.

### Data extraction and quality assessment

Two investigators independently extracted information from eligible studies using predesigned forms. The following data were extracted: first author’s name, year of publication, geographic location of study, sample size, age, treatment methods, study design, cut-off value, study end point, and HRs and 95% CIs for DFS and/or overall survival [OS]. Disagreements were resolved by consensus. The quality of the included studies was evaluated according to Newcastle-Ottawa Scale (NOS) [19]. The NOS contained three aspects: selection (4 points), comparability (2 points), and outcome assessment (3 points). Studies with a NOS score ≥6 were classified as high-quality studies.

### Statistical analysis

The impact of the NLR on DFS and/or OS was measured by pooled HRs and their 95% CIs extracted from included studies. If an HR and its 95% CI were not directly presented, they were computed by methods described by Parmar et al.[18]. Odds ratios (ORs) and 95% CIs were utilized to estimate the association between NLR and clinicopathological features. Heterogeneity among studies was evaluated using χ^2^-based Q test and Higgins’ *I*^2^ statistic. If *P*<0.10 and/or *I*^2^>50%, indicating significant heterogeneity among studies, the random-effects model (DerSimonian–Laird method) was used. Otherwise, the fixed-effects model (Mantel–Haenszel method) was applied. Publication bias was quantified using Begg’s funnel plot and the Egger’s linear regression tests. The dose-response meta-analysis was performed by using generalized least squares trend estimation, according to the methods developed by Greenland and Longnecker [20]. All statistical analyses were performed with Stata software version 12.0 (Stata Corp, College Station, TX). *P*<0.05 was considered statistically significant.

## Results

### Literature search

**Figure 1:**
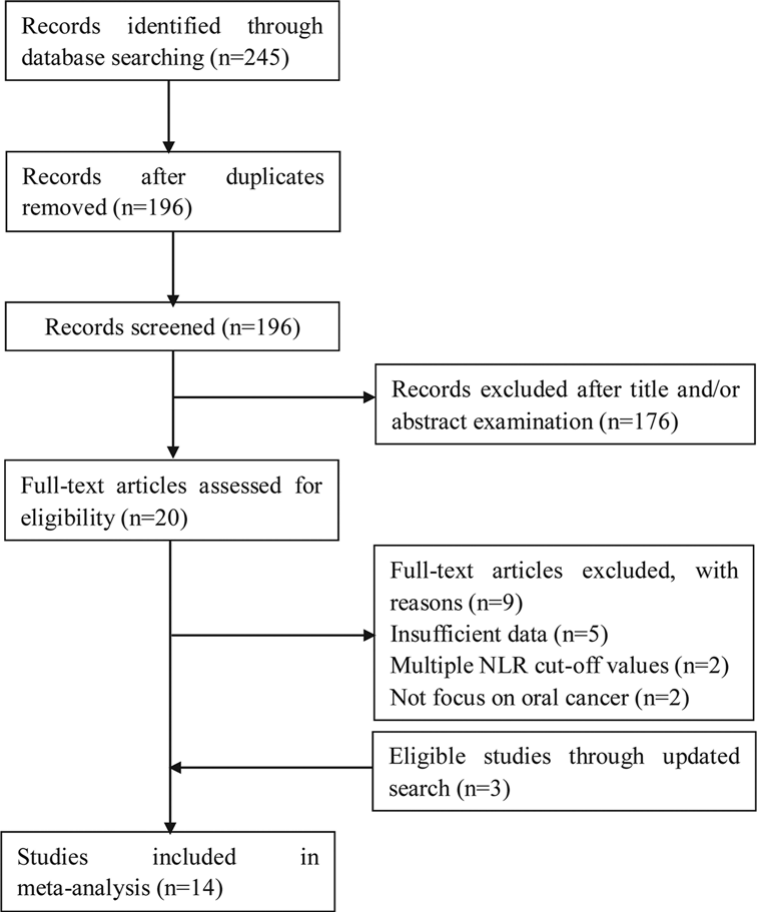
Flow chart of study selection process

A total of 245 records were retrieved by the electronic database search. After removing duplicates, 196 studies were identified. Of these, 176 records were removed via title and abstract examination and 20 full-text studies were checked. Subsequently, nine studies were excluded because of insufficient data, multiple cut-off values, or a focus other than oral cancer. Three eligible studies were identified through updated search. Ultimately, 14 studies comprising 3216 patients were included in the meta-analysis. The selection process is shown in Figure 1.

### Study characteristics

The basic characteristics of the included studies are shown in Table 1. The studies were published between 2013 and 2018 with sample sizes ranging from 68 to 613. All studies were retrospective. Twelve studies were conducted in Asia [11,13,14,16,21–28] and two were performed in Europe [12,15]. The cut-off values for the NLR ranged from 1.77 to 5. Ten studies [13–16,22–27] reported an association between the NLR and DFS, and nine studies [12,14,16,21–24,27,28] showed a correlation between the NLR and OS. Eight studies [11,15,16,21,23,24,26,28] presented data on the relationship between the NLR and clinicopathological factors. The NOS scores of the studies ranged from 6 to 8, with a median value of 7.

**Table 1:** Characteristics of all the studies included in this meta-analysis

Study	Year	Country/region	Number of patients	Study design	Age (years) Median/mean	Treatment	Study period	Cut-off value	NOS score
Acharya	2017	India	68	Retrospective	48.75	Surgery	2011–2014	1.77	7
Bobdey	2017	India	471	Retrospective	50	Mixed	2007–2008	2.38	8
Chen	2016	China	306	Retrospective	55	Surgery	2004–2009	2.7	7
Christina	2016	Austria	144	Retrospective	58	Chemoradiotherapy	2004–2014	1.9	7
Fang	2013	Taiwan	226	Retrospective	52.47	Surgery	2007–2012	2.44	7
Lee	2017	Taiwan	396	Retrospective	53	Surgery	2006–2013	2.73	8
Nakashima	2016	Japan	124	Retrospective	67.2	Mixed	2003–2009	2.4	7
Ong	2017	India	133	Retrospective	51.92	Surgery	2009–2013	1.88	7
Perisanidis	2013	Austria	97	Retrospective	NR	Mixed	2001–2009	1.9	6
Tsai	2014	Taiwan	213	Retrospective	53	Mixed	2004–2011	5	7
Wu	2017	Taiwan	262	Retrospective	51	Surgery	2004–2011	2.95	7
Park	2018	Korea	69	Retrospective	62	Mixed	2007–2016	2.29	7
Sano	2018	Japan	94	Retrospective	94	Surgery	2007–2014	2.36	8
Kao	2018	Taiwan	613	Retrospective	53	Surgery	2005–2014	2.28	8

Abbreviations: NOS, Newcastle-Ottawa Scale; NR, not reported.

### NLR and survival outcomes in oral cancer

**Figure 2:**
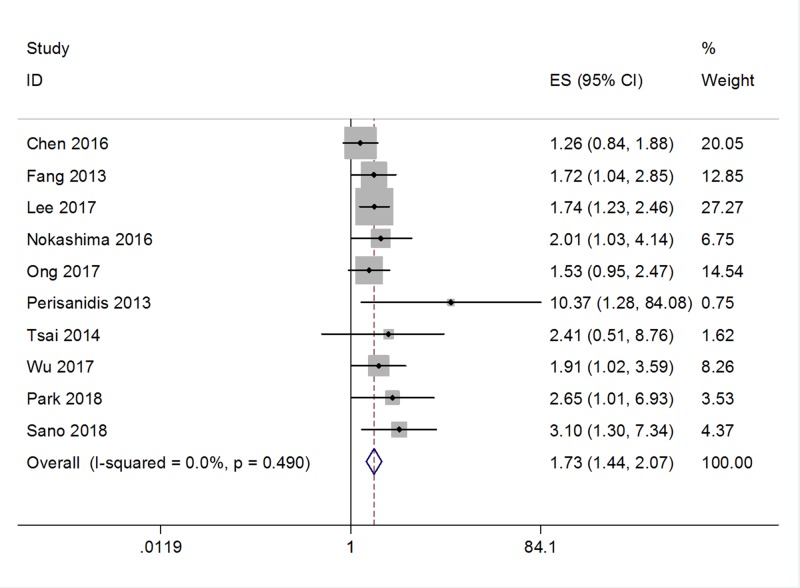
Forest plot depicting association between NLR and DFS in oral cancer

**Figure 3:**
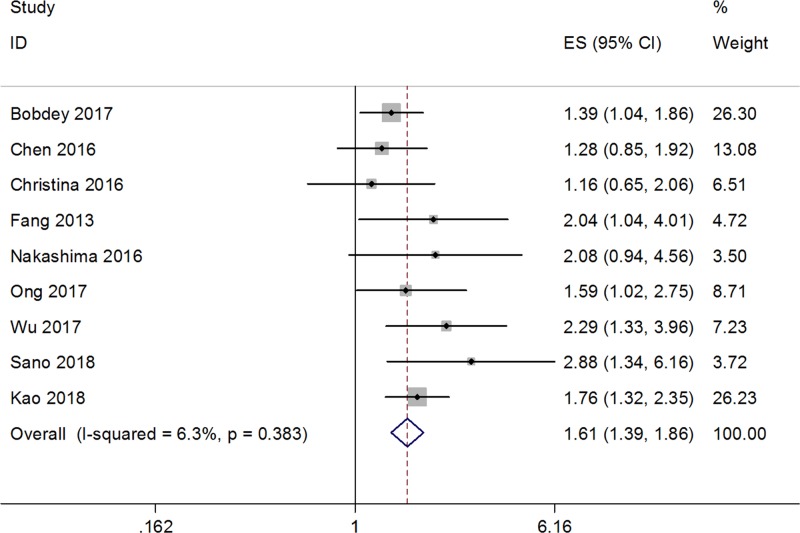
Forest plot depicting association between NLR and OS in oral cancer

Ten studies [13–16,22–27] with a total of 1920 patients evaluated the prognostic value of the NLR on DFS. No significant heterogeneity was detected (*I*^2^ = 0, *P*=0.49; Figure 2); therefore, a fixed-effects model was used. The pooled results indicated an HR of 1.73, with 95% CI = 1.44–2.07, and *P*<0.001. Next, data from nine studies [12,14,16,21–24,27,28] were merged to explore the impact of the NLR on OS. Significant heterogeneity was not observed (*I*^2^ = 6.3%, *P*=0.383; Figure 3), so a fixed-effects model was applied. The combined HR was 1.61, with 95% CI = 1.39–1.86 and *P*<0.001. Taken together, the pooled data demonstrated that a high NLR was associated with poor DFS and OS in oral cancer.

### NLR and clinicopathological characteristics in oral cancer

**Figure 4:**
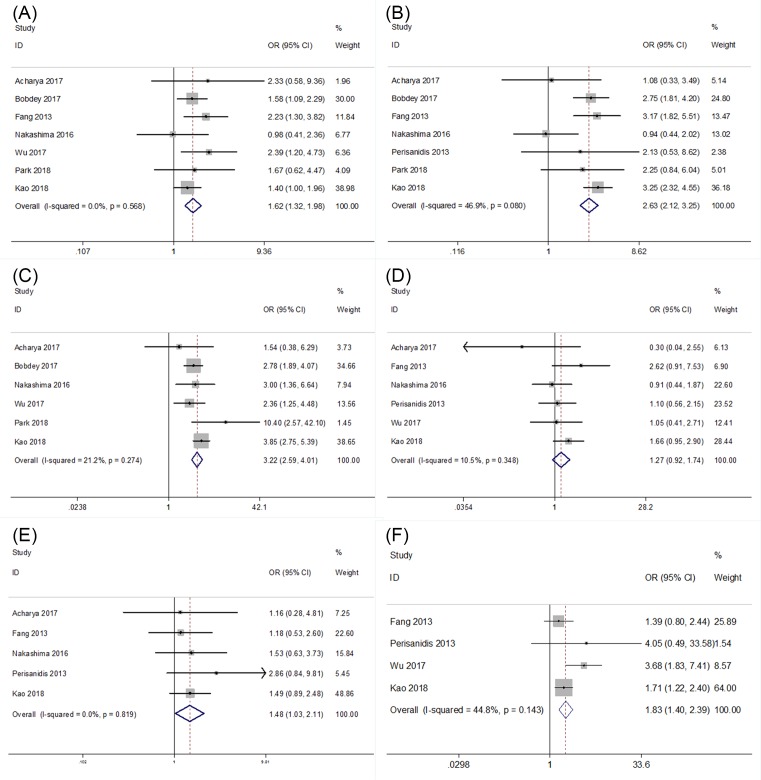
The relationship of the NLR and clinicopathological characteristics. Forest plots depicting correlations between NLR and (**A**) lymph node metastasis, (**B**) tumor stage, (**C**) T stage, (**D**) gender, (**E**) tumor differentiation, and (**F**) perineural invasion.

Ten studies [11,15,16,21,23,24,26,28] investigated the relationship of the NLR and clinicopathological characteristics, including lymph node metastasis, T stage, tumor stage, perineural invasion, gender and tumor differentiation. As shown in Figure 4 and Table 2, the results showed that a high NLR was significantly associated with lymph node metastasis (*n*=7, OR = 1.62, 95% CI = 1.32–1.98, *P*<0.001), advanced tumor stage (*n*=7, OR = 2.63, 95% CI = 2.12–3.25, *P*<0.001), T stage (*n*=6, OR = 3.22, 95% CI = 2.59–4.01, *P*<0.001), tumor differentiation (*n*=5, OR = 1.48, 95% CI = 1.03–2.11, *P*=0.033) and perineural invasion (*n*=4, OR = 1.83, 95% CI = 1.4–2.39, *P*<0.001). However, an elevated NLR was not correlated with gender (Figure 4 and Table 2).

**Table 2:** Meta-analysis of the association between NLR and clinicopathlogical factors in oral cancer

Features	Number of studies	OR (95% CI)	*P*	Heterogeneity		Effects model	Publication bias	
				*I*^2^(%)	*P*		Begg’s *P*	Egger’s *P*
Lymph node metastasis (positive vs negative)	5	1.76(1.36–2.28)	<0.001	0	0.452	FEM	0.806	0.858
T stage (T3-T4 vs T1-T2)	4	2.64(1.96–3.55)	<0.001	0	0.839	FEM	0.308	0.31
Tumor stage (advanced vs early)	5	2(1.22–3.27)	0.006	54.6	0.066	REM	0.624	0.274
Perineural invasion (positive vs negative)	3	2.36(1.07–5.22)	0.034	59.2	0.086	REM	1	0.676
Gender (male vs female)	5	1.11(0.75–1.63)	0.602	6.3	0.371	FEM	0.806	0.731
Differentiation (poor vs good/moderate)	4	1.46(0.89–2.41)	0.135	0	0.672	FEM	0.734	0.574
Lymph node metastasis (positive vs negative)	7	1.62(1.32–1.98)	<0.001	0	0.568	FEM	0.3	0.523
Tumor stage (advanced vs early)	7	2.63(2.12–3.25)	<0.001	46.9	0.08	FEM	0.23	0.095
T stage (T3-T4 vs T1-T2)	6	3.22(2.59–4.01)	<0.001	21.2	0.274	FEM	0.707	0.918
Gender (male vs female)	6	1.27(0.92–1.74)	0.146	10.5	0.348	FEM	0.707	0.428
Differentiation (poor vs good/moderate)	5	1.48(1.03–2.11)	0.033	0	0.819	FEM	0.806	0.709
Perineural invasion (positive vs negative)	4	1.83(1.4–2.39)	<0.001	44.8	0.143	FEM	0.734	0.446

Abbreviations: FEM, fixed-effects model; REM, random-effects model.

### Dose-response meta-analysis

Combined RRs comparing highest with lowest NLR were 2.34 (95% CI 1.49–3.25) for DFS, 2.73 (95% CI 1.62–4.01) for OS.

### Publication bias

**Figure 5:**
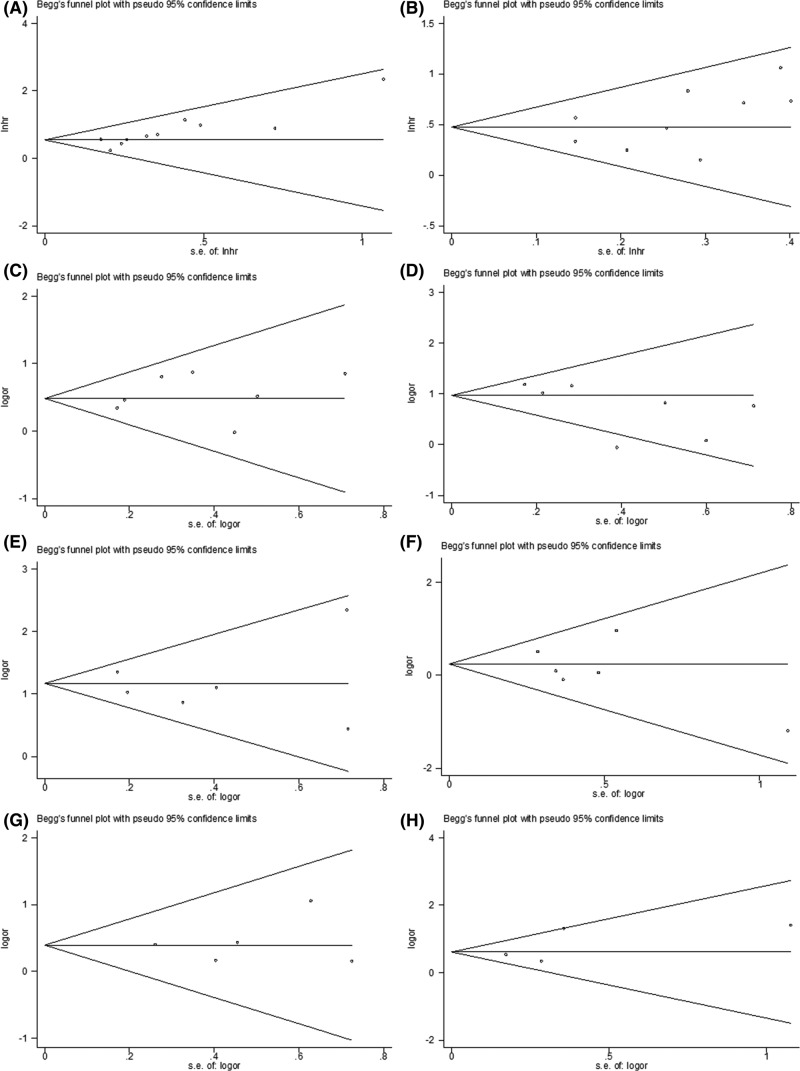
The potential publication bias estimation. Funnel plots on (**A**) DFS, (**B**) OS, (**C**) lymph node metastasis, (**D**) tumor stage, (**E**) T stage, (**F**) gender, (**G**) tumor differentiation, and (**H**) perineural invasion.

We used the Begg’s test and Egger’s test to estimate potential publication bias. The results of publication bias are listed in Table 2. The funnel plots are shown in Figure 5. All *P* values of publication bias were >0.05, suggesting that there was no evidence of publication bias in this meta-analysis.

## Discussion

This meta-analysis incorporating data from 14 studies showed that a high NLR was associated with poor DFS and OS in oral cancer patients. Furthermore, an elevated NLR was also significantly associated with lymph node metastasis, higher T stage, advanced tumor stage, tumor differentiation and perineural invasion. The present study collected the most recent data and provided relatively objective results concerning the prognostic role of the NLR in oral cancer.

Increased evidence indicates the important role of inflammation in tumorigenesis [29]. Due to the involvement of inflammatory responses in cancer, hematological parameters can provide important implications for cancer treatment and prognosis. Neutrophilia, a common occurrence in cancer patients, is often accompanied by relative lymphocytopenia [30]. Therefore, the NLR could be used as a simple index of systemic inflammatory responses in cancer patients. The NLR was first reported as a prognostic factor in colorectal cancer [31]. Walsh et al. found that a pre-operative NLR ≥5 was correlated with worse OS and cancer-specific survival in colorectal cancer patients receiving surgery [31]. Subsequently, the prognostic significance of the NLR was reported in various cancers [32]. With good accessibility and low cost, the NLR is a promising prognostic factor in daily practice. The biological validity underlying the NLR has also been investigated. In the tumor microenvironment, neutrophils can play a protumoral role by secreting matrix metalloproteinase 9 to facilitate carcinogenesis [33]. Moreover, neutrophils can also release factors that accelerate tumor cell proliferation [34]. Conversely, lymphocytes are known to impede malignant progression, and the infiltration of various subtypes of lymphocytes into tumor tissues has been demonstrated to predict increased survival in multiple cancers [35–37].

Other meta-analyses have also reported the prognostic value of the NLR in various cancers [38]. Chen et al. showed that the NLR was associated with poor OS in breast cancer [39]. Gu et al. reported that an elevated pretreatment NLR was correlated with both poor OS and PFS in non-small-cell lung cancer [40]. In addition, Tang et al. also demonstrated that a high NLR was significantly linked with detrimental long-term outcomes and clinicopathological parameters in patients with biliary tract cancer [41]. Our findings in the present study were in accordance with those of previous studies. It is noteworthy that a comprehensive meta-analysis including 100 studies explored the prognostic value of the NLR in various cancers [38]. That study was published in 2014, and only one study [15] on oral cancer was included. In the past several years, many new studies on the NLR and oral cancer have been published, and the current analysis contained 11 studies. Therefore, this is the most recent meta-analysis regarding the relationship between the NLR and oral cancer. A recent study [42] also investigated the association between the NLR and survival in oral cancer. That study conducted by Wang et al. [42] was performed well and concluded that a high NLR was correlated with poor DFS and OS in oral SCC, which was in accordance with our results. Our study also investigated the relationships between the NLR and clinical factors in oral cancer, whereas Wang’s study did not. We investigated the relationship of the NLR and clinicopathological characteristics, including lymph node metastasis, T stage, tumor stage, perineural invasion, gender and tumor differentiation. We found that the NLR was significantly associated with lymph node metastasis, T stage, advanced tumor stage, tumor differentiation and perineural invasion. These results provide implications for clinicians in practice. Therefore, our study was more comprehensive and provided more information on this issue.

However, there are several limitations to this meta-analysis. First, eligible studies used different cut-off values for the NLR; therefore, the definition of the high NLR group differed between studies. Second, most studies were conducted in Asia. Although this is in accordance with the high incidence of oral cancer in Asia, it may hinder the clinical use of the NLR in patients of other ethnicities. Third, confounders were not adjusted. Other pathological conditions such as cardiovascular disease, liver disease and infection can also influence the NLR. Because all eligible studies were retrospective cohort studies, the confounders may cause selection bias.

In conclusion, this meta-analysis showed that a higher NLR was associated with worse survival outcomes and several clinicopathological parameters in oral cancer. The NLR might be a potential independent prognostic factor in patients with oral cancer.

## Abbreviations

DFS: Disease-free survival
HR: Hazard ratio
NLR: neutrophil-to-lymphocyte ratio
NOS: Newcastle-Ottawa Scale
OS: overall survival
OR: odd ratio
RRs: Relative risks
SCC: squamous cell carcinoma
